# Characteristics of chest high resolution computed tomography images of COVID-19: A retrospective study of 46 patients

**DOI:** 10.12669/pjms.37.3.3504

**Published:** 2021

**Authors:** Yibo Lu, Jingru Zhou, Yimei Mo, Shulin Song, Xue Wei, Ke Ding

**Affiliations:** 1 Yibo Lu, Master of Medicine, Department of Radiology, Fourth People’s Hospital of Nanning, Nanning 530031, China; 2 Jingru Zhou, Master of Medicine, Department of Radiology, Fourth People’s Hospital of Nanning, Nanning 530031, China; 3 Yimei Mo, Bachelor of Medicine, Department of Radiology, Fourth People’s Hospital of Nanning, Nanning 530031, China; 4 Shulin Song, Master of Medicine, Department of Radiology, Fourth People’s Hospital of Nanning, Nanning 530031, China; 5 Xue Wei, Bachelor of Medicine, Department of Radiology, Third Affiliated Hospital of Guangxi Medical University, Nanning 530031, China; 6 Ke Ding, Doctor of Medicine, Department of Radiology, Third Affiliated Hospital of Guangxi Medical University, Nanning 530031, China

**Keywords:** COVID-19, Severe acute respiratory syndrome coronavirus 2, Tomography, X-Ray computed

## Abstract

**Objective:**

To analyze the characteristics of chest high resolution computed tomography (CT) images of coronavirus disease 2019 (COVID-19).

**Methods:**

This is a retrospective study analyzing the clinical records and chest high-resolution CT images of 46 consecutive patients who were diagnosed with COVID-19 by nucleic acid tests and treated at our hospitals between January 2020 and February 2020.

**Results:**

Abnormalities in the CT images were found in 44 patients (95.6%). The lesions were unilateral in eight patients (17.4%), bilateral in 36 patients (78.3%), single in seven patients (15.9%), and multiple in 37 patients (84.1%). The morphology of the lesions was scattered opacity in 10 patients (21.7%), patchy opacity in 38 patients (82.6%), fibrotic cord in 17 patients (37.0%), and wedge-shaped opacity in two patients (4.3%). The lesions can be classified as ground-glass opacity in eight patients (17.4%), consolidation in one patient (2.2%), and ground-glass opacity plus consolidation in 28 patients (60.9%).

**Conclusion:**

Most COVID-19 patients showed abnormalities in chest CT images and the most common findings were ground-glass opacity plus consolidation.

**Abbreviations:**
COVID-19: coronavirus disease 2019,
CT: computed tomography,
SARS-CoV-2: severe acute respiratory syndrome coronavirus 2,
RNA: ribonucleic acid.

## INTRODUCTION

Coronavirus disease 2019 (COVID-19) is an infectious disease caused by severe acute respiratory syndrome coronavirus 2 (SARS-CoV-2). The currently ongoing pandemic of COVID-19 is causing severe morbidity and mortality on a global scale. Its diagnosis is based on nucleic acid tests of the virus ribonucleic acid (RNA) using nasopharyngeal swabs.^1^,^2^ However, the diagnostic accuracy of RNA tests can be compromised by several factors, such as poor quality of the test kits, contamination of the swab samples, and inconsistent training and practice of the sampling staff.^3^-^5^ These factors are especially more common in developing countries and areas, resulting in great variation in the specificity of RNA tests ranging from 60% to 90%.^6^ In addition, asymptomatic patients of COVID-19 may have false-negative results in RNA tests.^7^ Considering these restrictions and limitations of RNA test, other diagnostic methods such as high-resolution computed tomography (CT) and X-ray should be considered for diagnosing COVID-19.^8^-^10^

The present study aimed to review the clinical data and the high-resolution CT images of COVID-19 patients and analyze the characteristics of the CT findings of this disease.

## METHODS

This is a retrospective study analyzing the clinical data and the CT images of 46 consecutive patients who were treated at our hospitals during January 24, 2020 and February 24, 2020. Our study was approved by the Ethical Committee of our hospital. The inclusion criteria were:

1.With symptoms of fever >38°C, cough, or dyspnea.
2.Positive results of SARS-CoV-2 RNA test using nasopharyngeal swabs.

Patients with the following conditions were excluded:

1.Pregnancy.
2.No CT examinations.
3.Patients who were transferred to other hospitals or were lost to follow-up.

### CT scan:

All patients received high-resolution CT scans at admission and 1–5 days later using a 64-slice CT scanner (Optima CT680, GE, USA). The scanning scope was between the superior aperture of the thorax and the posterior costophrenic angle with a slice interval of 1 mm and a slice thickness of 1.25 mm. The voltage was 120 kV. The current was 100–420 mA with automatic adjustment. The mediastinal window level was 40 HU with a window width of 400 HU. The lung window level was -500 HU with a window width of 1500 HU. All CT images were analyzed by three senior radiologists. Consensus was reached by discussion.

### Treatment:

Most of our patients had mild illness and were treated with antiviral drugs (oseltamivir, Aluvia, interferon, and arbidol) in 46 patients (100%), antibiotics (moxifloxacin, teicoplanin, and cefoperazone plus sulbactam) in 12 patients (26.1%), and methylprednisolone in seven patients (15.2%). Criteria for discharge included:

1.No fever for at least three days.
2.No respiratory symptoms.
3.Significant improvement in lung effusions shown by CT.
4.Negative results of two consecutive RNA tests spaced by at least 24 hours.

## RESULTS

A total of 46 patients were included. There were 21 males (45.7%) and 25 females (54.3%). The mean age was 45.2 years. Twenty-five patients had recently been in Wuhan city or in close contact with people who were from Wuhan city. The general characteristics and comorbidities of our patients are shown in Table-I.

**Table-I:** Patient general characteristics and comorbidities.

Characteristics or comorbidities	
Male (n)	21 (45.7%)
Age (year, mean)	45.2
Age (year, median and range)	46 (5–73)
Type 2 diabetes (n)	3 (6.5%)
Hypertension (n)	8 (17.4%)
Hepatitis B (n)	1 (2.2%)
Lupus (n)	1 (2.2%)
Stroke (n)	1 (2.2%)
Coronary heart disease (n)	1 (2.2%)

### Characteristics of CT images:

Most of our patients showed abnormalities in chest CT (44/46, 95.7%). The characteristics of chest CT images are shown in Table-II.

**Table-II:** Characteristics of chest CT images.

	N = 46	%
Lesion distribution	7	15.2
Single lesion	37	80.4
Multiple lesions	8	17.4
Unilateral	7	15.2
Bilateral	36	78.3
No abnormality	2	4.3
*Lesion morphology*	
Scattered opacity	10	21.7
Patchy opacity	38	82.6
Fibrotic cord	17	37.0
Wedge-shaped opacity	2	4.3
*Lesion density*	
Ground-glass opacity	8	17.4
Consolidation	1	2.2
Ground-glass opacity plus consolidation	28	60.9
None	6	13.0
*Accompanying abnormality*	
Interlobular septal thickening	12	26.1
Intralobular septal thickening	29	63.0
Air bronchogram	27	58.7
Bronchovascular thickening	30	65.2
Bilateral pleural thickening	4	8.7
Pleural effusion	1	2.2

### Treatment results and typical cases:

The mean hospital stay was 8.2 days. Viral RNA was negative in two consecutive tests in 30 patients (65.2%) after treatment of 4–17 days. All patients finally met the discharge criteria and no death occurred. Improvement in lung lesions shown by CT was noticed in 40 patients (87.0%) during the first week of hospitalization, in 22 patients (62.9%) during the second week of hospitalization, and in eight patients (66.7) during the third week of hospitalization.

### Case-1:

**Fig.1:**
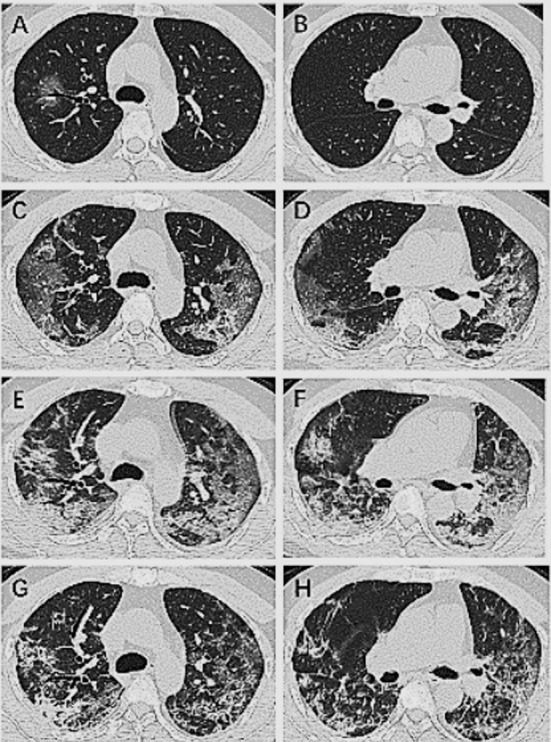
Chest CT images of a 52-year-old man with COVID-19 who was treated for 17 days.

A 52-year-old man with COVID-19 was treated for 17 days and had negative results in two consecutive viral RNA tests. On the first day at hospital, chest CT showed scattered ground-glass opacities with air bronchogram and bronchovascular thickening (Fig.1AB). On the sixth day, the lesions expanded, and consolidations were seen in the ground-glass opacities (Fig.1CD). On the 13^th^ day, the lesions continued to expand, and more consolidations appeared (Fig.1EF). On the 18^th^ day, both the consolidations and the ground-glass opacities were reduced (Fig.1GH).

### Case-2:

**Fig.2:**
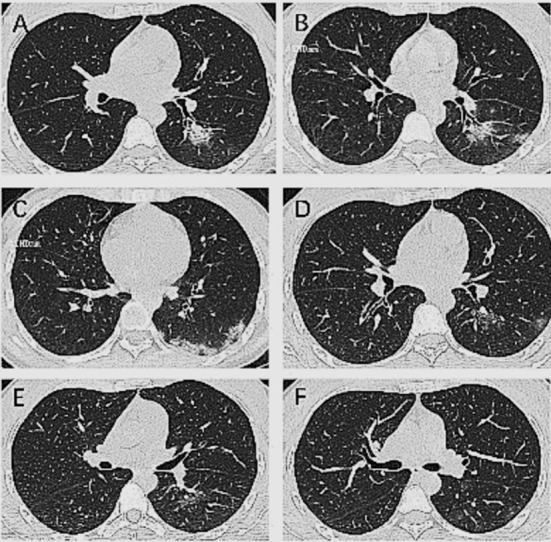
Chest CT images of a 25-year-old woman who was treated for COVID-19 for 14 days.

A 25-year-old woman was treated for COVID-19 for 14 days and had negative results in two consecutive viral RNA tests. On the 1^st^ day at hospital, chest CT showed a consolidation opacity in the lower lobe of the left lung (Fig.2A). On the 5^th^ day, the consolidation opacity expanded, subpleural consolidations were seen with surrounding halo signs (Fig.2BC). On the 10^th^ day, the consolidation opacity in the lower lobe of the left lung showed reduced density and size (Fig.2D). One the 13^th^ day, this consolidation was absorbed with residual scattered ground-glass opacities (Fig.2EF).

### Case-3:

**Fig.3:**
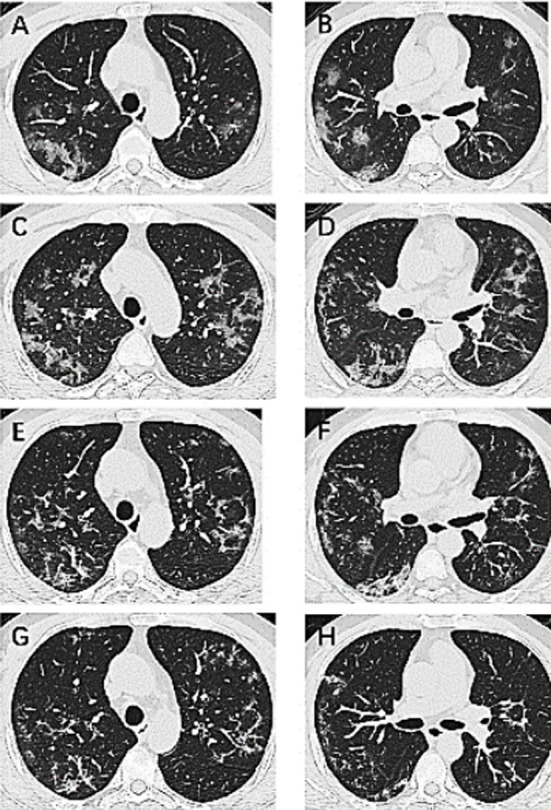
Chest CT images of a 45-year-old man with COVID-19 who was treated for 13 days.

A 45-year-old man with COVID-19 was treated for 13 days and had negative results in two consecutive viral RNA tests. On the first day at hospital, chest CT showed bilateral subpleural ground-glass opacities with bronchovascular thickening (Fig.3AB). On the third day, there were more ground-glass opacities with reversed halo signs (Fig.3CD). On the 6^th^ day, the lesions on the CT images decreased (Fig.3EF). On the 11^th^ day, most of the lesions were absorbed with residual patchy, cord opacities (Fig.3GH).

### Case-4:

**Fig.4:**
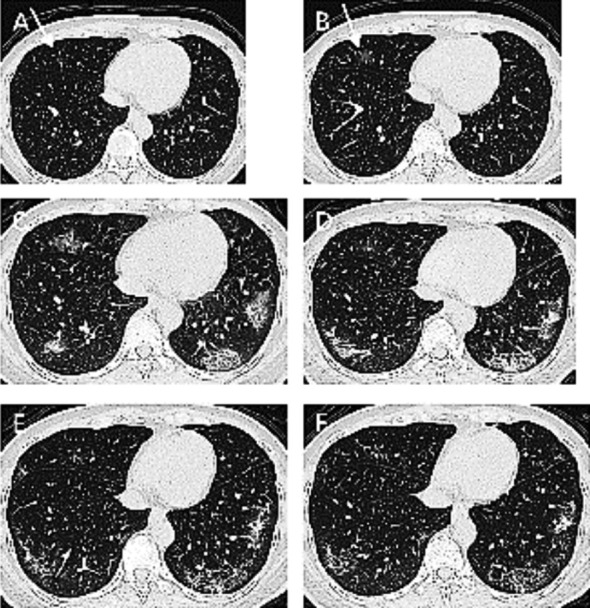
Chest CT images of a 49-year-old woman with COVID-19 who was treated for 16 days.

A 49-year-old woman with COVID-19 was treated for 16 days and had negative results in two consecutive viral RNA tests. On the first day at hospital, chest CT found a single ground-glass opacity in the middle lobe of the right lung (arrow, Fig.4A). On the second day, the lesion expanded with increased density (arrow, Fig.4B). On the 6^th^ day, this lesion continued to grow, and subpleural ground-glass opacities appeared in the bilateral lower lobes with consolidations and intralobular septal thickening (Fig.4C). On the 12^th^ day, the lesion in the middle lobe of the right lung decreased in size and density, while the lesions in the bilateral lower lobes increased in size and density (Fig.4D). On the 17^th^ day, the consolidations in the bilateral lower lobes decreased (Fig.4E). On the 21^st^ day, the lesions were largely absorbed (Fig.4F).

## DISCUSSION

Our study retrospectively analyzed the chest CT images of 46 patients with COVID-19 and found that most of them showed abnormalities in CT images. The lesions on chest CT change alone with the clinical course of COVID-19 and reflect the disease severity and patient recovery.

SARS-CoV-2 belongs to the β-coronavirus genus and results in lung pathologies like that of SARS and Middle East respiratory syndrome.^11^ The most prominent pathology in SARS is lung lesions. The virus can directly infect the lung tissues and cause injury. Other mechanisms are responsible for the secondary injuries in SARS, one of which is the shedding of epithelial cells of the alveoli and the bronchi, leading to obstruction in these structures.^12^ The radiological feature of viral pneumonia is multifocal lesions that distribute along the bronchovascular structures and under the pleura.^13^

Most of our patients (42/46) had mild symptoms of COVID-19 and 44 patients (95.6%) had abnormalities in chest CT. This finding suggests that early CT examination may be useful in diagnosing COVID-19 patients who are asymptomatic or mildly symptomatic. The early findings in the chest CT images of COVID-19 were ground-glass opacity plus consolidation, and only very few patients had either change alone. The primary pathology in the lung is inflammatory effusion, including pulmonary edema, focal lung cell proliferation, and inflammatory cell infiltration.^14^-^16^ The corresponding CT findings are bronchovascular thickening and air bronchogram. Along with more effusion and larger and denser lesions, chest CT may show consolidation opacities, which conceal the vessels and the bronchi in the lesions. COVID-19 pneumonia may primarily show intralobular septal thickening and occasional interlobular septal thickening. When both signs are present, the crazy-paving pattern may appear. SARS-CoV-2 mostly attacks the deep airways, results in inflammatory reactions and injury to the alveolar epithelium. The lesions are usually peripheral and subpleural. In our study, 80.4% (37/46) of the patients had multifocal lesions involving bilateral lungs and multiple lobes and segments. Single lesions were found in 15.2% (7/46) of the patients, which were focal and located in one segment. Pleural effusion, mediastinal lymphadenopathy, and extrapulmonary lesion were rare in our patients

CT examinations within one week of hospitalization showed that the lung lesions were absorbed in 26 (65%) patients and increased in 14 (35%) patients. This finding suggests a rapid development or recovery of COVID-19 and CT examination within one week after treatment is useful in evaluating patient status. After treatment of two to three weeks, chest CT of most patients showed absorption of the lesions or no significant change of the lesions. At this stage, the disease was stabilized and gradually progressed into the absorption phase of the lesions. Only a few patients showed more lesions in CT images. These patients were generally older and had more comorbidities, which might delay the absorption of the lesions. Notably, 14 patients were followed-up with chest CT scans after discharge. These CT images showed residual cord opacities and ground-glass opacities. These patients were still being observed to find out whether these lesions will be absorbed eventually or exist for a longer time.

Due to many affecting factors, such as the sampling technique, test kit quality, and nature of the disease, the false-negative issue in SARS-CoV-2 RNA tests is still a concern.^17^,^18^ Among the 44 patients with chest CT abnormalities at the first examinations, 10 (22.7%) patients required at least two tests of viral RNA to confirm the diagnosis of COVID-19. This indicates the possibility of false-negative results in viral RNA tests. Thus, a chest CT scan may provide a backup diagnostic method for potentially infected patients who have evidence of close contact with confirmed patients.

### Limitations:

Our study has limitations. First, the follow-up time was relatively short and the final imaging results of the fully recovered patients were not all obtained. Second, the treatment may have some effects on the disease process of COVID-19.

## CONCLUSION

Most COVID-19 patients show characteristic features in high-resolution CT images even from the early stage of the infection. The most common chest CT findings are ground-glass opacity plus consolidation. Chest CT is an optional diagnostic method for potentially infected patients who may have false-negative results in SARS-CoV-2 RNA tests. High-resolution CT examination can also provide useful information for evaluating treatment efficacy.

### Authors’ Contribution:

**YL and XW** collected the data.

**JZ, YM, and SS** analyzed the data.

**KD and YL** conceived the study and drafted the manuscript and these authors have equal contributions to this work.

**KD** is responsible and accountable for this study.
